# Efficient acquisition of tens of thousands of short tandem repeats in single-cell whole-genome-amplified DNA

**DOI:** 10.1016/j.xpro.2021.100828

**Published:** 2021-09-16

**Authors:** Liming Tao, Zipora Marx, Ofir Raz, Ehud Shapiro

**Affiliations:** 1 Department of Computer Science and Applied Mathematics, Weizmann Institute of Science, Rehovot 761001, Israel

**Keywords:** Single Cell, Genomics, Sequencing, High Throughput Screening, Molecular Biology, Molecular/Chemical Probes

## Abstract

Short tandem repeats (STRs) are highly abundant in the human genome, but existing approaches for accurate genotyping of STRs are limited. Here, we describe a protocol for duplex molecular inversion probes for high-throughput and cost-effective STR enrichment. We have successfully tested panels targeting as many as 50K STRs in several thousands of genomic samples (e.g., HeLa cells, Du145 cells, leukemia cells, melanoma cells). However, because the protocol is plate based, the sample size is limited to a few thousand.

For complete details on the use and execution of this protocol, please refer to Tao et al. (2021).

## Highlights

• This protocol enable us to enrich tens of thousands of STR from single-cell WGA
• The protocol can easily deployed in any labs with generic equipment
• The highly mutable STR panel is generic across human samples
• The panels are highly customizable to include SNV targets like cancer hotspots and flexible from dozens of targets to over 50K targets; probes for different panels can be combined in one reaction

## Graphical abstract

**Figure:**
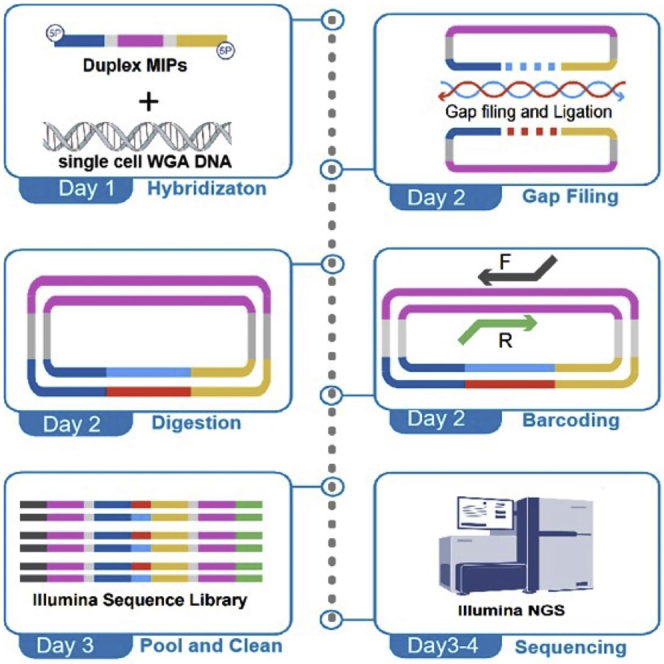

## Before you begin

The protocol below describes the specific steps for using whole genome amplified genomic DNA (REPLI-g Mini Kit, Qiagen) from Du145 single cells for the 12K OM6 STR panel presented in our Cell Reports Methods paper (Tao et al., 2021) (Custom Array). However, we have also used this protocol for primary cells such as melanoma, leukemia, T-cells, Macrophages, *etc.* and other whole genome amplification kits such as REPLI-g Single Cell Kit, Ampli1WGA kit, MALBAC single cell WGA kit etc.

### Duplex MIP preparation

**Timing: [2 days]**

Prepare the duplex molecular inversion probes for a 12K panel of selected human STRs, OM6, to enrich these targets from the single cell WGA DNA in the following steps.

1. KOD Hot Start Real Time Custom PCR Mix 5**×** (KOD 5**×** Custom Mix)
  a. Prepare SYBR 100**×** by mixing 10 μL from stock SYBR green I (Lonza, 10,000**×**) and 990 μL Dimethyl Sulfoxide (DMSO) (Sigma).
  b. Prepare 2 mL KOD 5**×** Custom Mix according to the table below.

**Table:** 

Reagents	Stock conc.	Final conc.	KOD 5**×** custom mix (μl)
ddH_2_O			0.27
KOD Buffer 10**×** (Merck)	10**×**	5**×**	2.5
MgSO_4_ 25 mM (Merck)	25 mM	7.5 mM	1.5
dNTP 25 mM each (Bioline)	25 mM	7.5 mM	0.2
KOD Enzyme 1 U/μL (Merck)	1 U/μL	0.1 U/μL	0.5
SYBR 100**×** (Lonza)	100**×**	1**×**	0.025
Total Volume			5

2. PreAmp PCR (8 reactions)
  a. Dilute the synthesized oligo pool (Custom Array, Inc.) to 1 ng/μL to prepare PCR template.
  b. Amplification primers designed to bind universal adapters are used for PreAmp PCR in LightCycler 480 (LC480, Roche) as shown below:
    OM4_Mly_F: GTCTATGAGTGTGGAGTCGTTGC
    OM4_Mly_R: CTAGCTTCCTGATGAGTCCGATG
    ***Note:*** SYBR in KOD 5**×** Custom Mix can be used to track the amplification for real time PCR.
    PreAmp PCR Mix:

**Table:** 

Reagents	Stock conc.	Final conc.	1**×** PreAmp PCR mix (μl)
Template	1 ng/μL	0.2 ng/μL	1.8
OM4_Mly_F primer	10 pmol/μL	0.3 pmol/μL	1.35
OM4_Mly_R primer	10 pmol/μL	0.3 pmol/μL	1.35
KOD 5**×** Custom Mix	5**×**	1**×**	9
ddH_2_O			31.5
Total Volume			45
    PreAmp PCR program:

**Table:** 

PCR cycling conditions			
Steps	Temperature	Time	Cycles
Initial Denaturation	95°C	120 s	1
Denaturation	95°C	20 s	18 cycles
Annealing	60°C	10 s	
Extension	70°C	5 s	
Final extension	70°C	50 s	1
Hold	4°C	Forever
  c. Purify PreAmp PCR product by MinElute PCR purification kit (Qiagen).
  d. Measure concentration by Qubit dsDNA HS Assay Kit (Life Technologies).

3. Production PCR (48 reactions). Troubleshooting 3
  a. Dilute purified PreAmp PCR product to 1 ng/μL for template.
  b. 96 well plate production PCR is performed according to the setup below. Amplification is tracked by SYBR present in the KOD 5**×** Custom Mix.

**Table:** 

Reagents	Stock conc.	Final conc.	1**×** production PCR (μl)
Template	1 ng/μL	0.2 ng/μL	1.8
OM4_Mly_F primer	10 pmol/μL	0.3 pmol/μL	1.35
OM4_Mly_R primer	10 pmol/μL	0.3 pmol/μL	1.35
KOD 5**×** Custom Mix	5**×**	1**×**	9
ddH_2_O			31.5
Total Volume			45
    Production PCR program

**Table:** 

PCR cycling conditions			
Steps	Temperature	Time	Cycles
Initial Denaturation	95°C	120 s	1
Denaturation	95°C	20 s	12 cycles
Annealing	60°C	10 s	
Extension	70°C	5 s	
Final extension	70°C	50 s	1
Hold	4°C	Forever
  c. PCR product are pooled and purified by MinElute columns (Qiagen).
  d. Elute with 45 μL ddH_2_O per column.
  e. Pool all purified products.
  f. Measure the DNA concentration of the final pool by loading 1 μL of the pool onto a NanoDrop spectrophotometer (Thermo Scientific).
  g. Dilute the pool to ∼30 ng/μL based on measured concentration.
  h. Retain 20 μL of sample to evaluate size distribution in Step 6. Carry the rest forward in Step 4.

4. Digest the diluted DNA. Troubleshooting 4
  a. Combine diluted DNA with MlyI following the table below

**Table:** 

Reagents	Stock conc.	Final conc.	1**×** with MlyI mix (μl)
Diluted DNA (30 ng / uL)	30 ng/μL	25.2 ng/μL	84
10**×** NEB Smarter Buffer	10**×**	1**×**	10
MlyI	10 U/μL	0.6 U/μL	6
Total Volume			100
  b. Incubate the mixture at 37°C overnight, deactivate at 80°C for 20 min, and store at 4°C.

5. Prepare final duplex MIP pool.
  a. Purify digested DNA by MinElute column.
  b. Pool elution samples into one tube.
  c. Measure concentration using by Qubit dsDNA HS (High Sensitivity) assay kit according to the manufacturer's protocol.
6. Perform quality control on digested product size distribution. Run digested and undigested samples (Step 4b) on Tape Station (Agilent). The final duplex MIP pool should be ∼105 bp, and undigested sample from step (4b) should be ∼150 bp. (Figure1).

**Figure 1:**
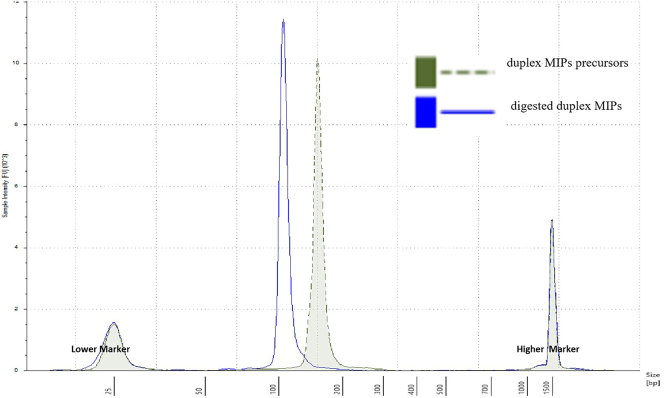
Duplex MIPs quality control
7. Based on length of 105 bp and the concentration, the final duplex MIPs pool is diluted to 80 nM (80 fmol/μL) stock solution, equivalent to 5.8 ng/μL. Dilute further to 8 nM as working solution. Store both stock and working solutions at −20°C.

### Whole-genome-amplified genomic DNA preparation

**Timing: [15 min]**

Single-cell WGA DNA is prepared by selected kit in advance. Here we just describe thawing of the single cell WGA DNA for the following step.

8. Clean the bench with 70% Ethanol. Take out a plate of whole genome amplified genomic DNA from −20 freezer.
9. Thaw at room temperature.
10. Shake on a bench top mixer, quickly spin down (approximately 30 s) at 500 rpm.

**CRITICAL:** Keep the plate well sealed to avoid cross contamination.

## Key resources table

**Table:** 

REAGENT or RESOURCE	SOURCE	IDENTIFIER
**Chemicals, peptides, and recombinant proteins**		

Betaine solution	Sigma	Cat#5MB0306 1VL
KOD enzyme	Merck	Cat# 71086
dNTP Set	Bioline	Cat#BIO-39049
SYBR 100**×**	Lonza	Cat#50513
Phusion High-Fidelity DNA Polymerase	NEB	Cat#NEB-M0530L
Ampligase 10**×** Reaction Buffer	Epicentre	Cat#A1905B
Ampligase DNA Ligase W/O Buffer	Epicentre	Cat#A3210K
Exonuclease I (*E. coli*)	NEB	Cat#M0293L
Exonuclease III (*E. coli*)	NEB	Cat#M0206L
RecJf	NEB	Cat#M0264L
Exonuclease T	NEB	Cat#M0265L
T7 Exonuclease	NEB	Cat#M0263L
Lambda Exonuclease	NEB	Cat#M0262L
NEBNext Ultra II Q5 MasterMix	NEB	Cat#M0544L
MinElute PCR Purification Kit	QIAGEN	Cat#28006
Qubit® dsDNA HS Assay Kit	Thermo Fisher	Cat#Q32854
Agencourt Ampure XP Beads	Beckman Coulter	Cat#A63881
2% Agarose, dye-free, BluePippin, 100–600,	Sage	Cat#BDF2010
TapeStation ScreenTape	Agilent	Cat#5067-5582
TapeStation Reagents	Agilent	Cat#5067-5583
MiSeq Reagent Kits v2	Illumina	Cat#MS-102-2002
MiSeq Reagent Nano Kit v2 (300-cycles)	Illumina	Cat#MS-103-1001
NextSeq 500/550 High Output Kit v2.5 (300 Cycles)	Illumina	Cat#20024908

**Deposited data**		

Sequencing data	ArrayExpress	E-MTAB-6411

**Experimental models: cell lines**		

DU145 cell line	ATCC	DU 145ATCC® HTB-81™

**Oligonucleotides**		

Oligopool	GenScript	OM6(Tao et al., 2021)

## Step-by-step method details

### STR target enrichment

**Timing: [2 days]**

In this step, we enrich all the designed targets from every single cell WGA DNA in 96 well plates.

1. Hybridization
  a. Make Hybridization Mix with 200–500 ng of single cell WGA DNA (∼2 μL) per reaction. Note that single cell WGA product concentration is generally 100–200 ng/μL in our hands.
    i. For large scale experiments, prepare Hybridization Master Mix according to the following table without WGA DNA. Distribute 8μL Hybridization Master Mix per well of a 96-well plate. Add 2 μL DNA or ddH_2_O to each well and mix by liquid handling system (Evoware, Tecan) or manually.

**Table:** 

Reagents	Stock conc.	Final conc.	1**×** hybridization mix (μl)
Single Cell WGA DNA	100 ng/μL	20 ng/μL	2
Duplex MIPs	8 fmol/μL	0.8 fmol/μL	1
Ampligase Buffer	10**×**	1**×**	1
Betaine	5M	0.9 M	1.8
ddH_2_O			4.2
Total Volume			10
  b. Place the reaction plate into a PCR machine with 100°C lid temperature. Heat at 98°C for 3 min and ramp the temperature at 0.01°C per second to 56°C.Then, incubate at 56°C for 17 h. An example in our PCR machine is shown below.

**Table:** 

Step	Temperature	Time	Cycles
1	97.9°C	3 min	
2	97.9°C	15 s	**×**420
	decrease as slow as 0.1ºC/sec		
	decrease by 0.1°C/sec every cycle		
3	56°C	17 h	
4	56°C	Pause for adding gap filing mix

2. Gap filling
  a. Prepare Gap Filling Mix half an hour before hybridization finishes. See table below.

**Table:** 

Reagents	Stock con.	Final conc.	1**×** gap filling Mix(μl)
dNTP	2 mM	0.3 mM	1.5
NAD	10 mM	2 mM	2
Betaine	5M	1.1 M	2.2
Ampligase buffer	10**×**	1**×**	1
Ampligase	5 U/μL	0.5 U/μL	1
Phusion	2 U/μL	0.8 U/μL	0.4
ddH_2_O			1.9
Total Volume			10
  b. Keep the mix at 56°C on a heat block
  c. Transfer reaction plate from the PCR machine to a 56°C heat block when the hybridization step is finished.
  d. Add 10 μL of Gap Filling Mix to each well, carefully mix by pipette, seal tightly and quickly return plate to the PCR machine.
  e. Run a 4-h 56°C incubation, deactivate for 20 min at 68°C, then keep at 4°C until next step.

**Pause point:** After the gap filling step, the reaction plate can be stored at 4°C fridge for up to two days.

3. Digestion of linear DNA:
  a. Prepare Digestion Mix 15 min before gap filling ends.

**Table:** 

Reagents	Stock con. (U/μL)	Final conc. (U/μL)	1**×** digestion mix (μl)
exo I	20	3.5	0.175
exo III	100	18	0.18
exo T7	10	4	0.4
exo T	5	0.4	0.08
RecJf	30	3	0.1
lambda exo	10	0.2	0.02
ddH2O			1.045
Total Volume			2
  b. Retrieve reaction plate from PCR machine. Note: take care when removing cover.
  c. Add 2 μL of the Digestion Mix to each well and mix.
  d. Spin down the reaction plate and seal.
  e. Incubate at 37°C for 60 min, 80°C for 10 min and 95°C for 5 min.

**Pause point:** the reactions can be stored at −20°C for at least 2x months after the digestion step.

**CRITICAL:** Seal the plate tight, avoid evaporation.

### Library preparation and sequencing

**Timing: [4 days]**

Illumina sequencing adapters and unique barcode per cell are added by a barcoding PCR. Then all the samples are pooled into one tube in equal volume and then equal molecular concentration. The pools are size selected by Blue Pippin to remove dimmers and by products. library pools passed quality control are sequenced on MiSeq or NextSeq with default illumine sequencing primers.

4. Sample specific barcoding PCR
  a. Note the structure of the dual-index Illumina barcoding primers used in the experiments:
    i. i5-index-primer: AATGATACGGCGACCACCGAGATCTACAC[i5-8bp-index]ACACTCTTTCCCTACACGACGCTCTTCCG;
    ii. i7-index-primer: CAAGCAGAAGACGGCATACGAGAT[i7-8bp-index]GTGACTGGAGTTCAGACGTGTGCTCTTCCG;
  b. 2 μL product from the previous step (step 3) are amplified with a pair of unique barcoding primers for each sample in a reaction as shown below.

**Table:** 

Reagents	Stock conc.	Final conc.	1**×** (μl)
Template	NA	NA	2
dual-index Illumina primers	5 pmol/μL each	0.5 pmol/μL each	2
NEBNext Ultra II Q5 Master Mix	2**×**	1**×**	10
SYBR 100**×**	10**×**	0.5**×**	1
ddH2O			5
Total Volume			20

**Table:** Barcoding PCR program

Temperature	Time	Cycles
98°C	30 s	
98°C	10 s	**×**5 cycle
56°C	30 s	
65°C	45 s	
98°C	10 s	**×**15 cycle
65°C	75 s	
65°C	5 min	
4°C	Hold

**Figure 2:**
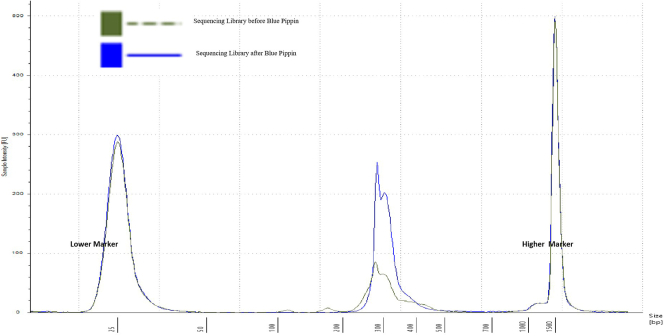
Quality control of sequencing library
  f. Dilute size-selected pool to make 12 μL of 4 nM (4 fmol/μL) library for Illumina NGS calculated based on the concentration and average size reported by the Tape Station.

**Figure 3:**
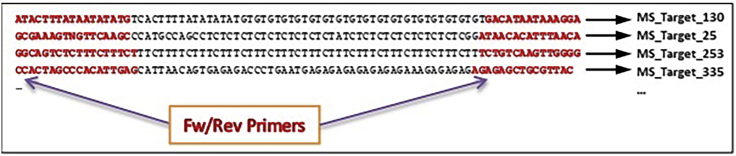
MS reads mapping Each read is mapped to a specific target locus according to its flanking regions.
  d. For more details, parallel execution and integration to the clineage analysis system, please see the codes at: https://github.com/shapirolab/clineage/blob/master/sequencing/analysis/full_msv/full_msv.py
  e. Extract the total number of reads per sample from “sorted_assignment_bam” with pysam.

5. Sample pooling and Purification for Diagnostic Sequencing
  a. Clean up barcoded PCR product in a 96-well plate using 0.8**×** AMPure XP SPRI magnetic beads (Beckman Coulter) according to manufactory’s manual by Tecan liquid handling system, eluted in 40 μL ddH2O.
  b. Pool equal volumes (usually take 2 ul) of purified samples manually.
  c. Concentrate the pool by MinElute according to manufacturer instructions, elute with 35 μL ddH2O.
6. Size Selection for Diagnostic Sequencing
  a. Retain 3 ul of the concentrated pool for quality control in step 5.
  b. Run 30 μL of the concentrated pool on a lonza 2% V1 cassette BluePippin (Sage Science) with setting range 240–340 bp according to manufactory’s protocol. Agarose gel extraction in the range of 240–340 bp can serve as an alternative.
  c. Purify size-selected elution by MinElute, elute with 15 μL ddH2O.
  d. Measure concentration by Qubit dsDNA HS (High Sensitivity) assay kit. Troubleshooting 1
  e. Inspect size distribution of the concentrated pool before and after size selection using a Tape Station dsDNA chip (Figure 2 is a reuse of panel 1 in Supplementary Figure 1 from our *Cell Reports Methods* paper (Tao et al., 2021) and confirms a single peak around 300 bp. Troubleshooting 2

7. Diagnostic sequencing (∼17 h for sequencing, ∼2 h for analysis) Troubleshooting 5
  a. Sequence at 10 pM loading concentration. We recommend to run on a 300 cycle MiSeq Nano flow cell in pair end mode. Set Read1 and Read2 as 151, and both Index1 and Index2 reads as 8. Minimum read length we have tested is 125 **×** 2 pair end to allow sequencing through the repeat regions of most STRs in our design. Default sequencing primers suffice for sequencing.
  b. Following bcl2fastq demultiplexing, merge overlapping Read1 and Read2 with the following command:
     >pear -v 40 -m 300 -f fastq1 -r fastq2 -o
    pear_files_prefix
  c. Map merged reads against customized STR reference (as shown in Figure 3) of all amplicons with bowtie2, each appearing multiple times, once with every possible STR length.
     >bowtie2 -x index_files_prefix -U merged_fastq |
    samtools view -bS - | samtools sort -o
    sorted_assignment_bam

8. Balancing reads per sample
  a. Calculate the scaling volume for each sample based on the total number of reads extracted from the diagnostic sequencing result to equalize the read coverage per sample. For example, sample A got 500 reads, sample B got 1000 reads in the diagnostic sequencing, to equalize the read coverage in the following production sequencing, we can pool 2 ul sample A with 1 ul sample B.
  b. According to the scaling volume, pool purified samples from step (5a) manually or by Echo550, then concentrate by miniElute, elute in 35μL ddH2O.
  c. Prepare production sequencing library for pooled samples as in step (6).
9. Production sequencing (∼29 h for sequencing)

The minimum reads per samples is 1M, and the minimum read length is 125 **×** 2 pair end. We recommend to sequence up to 200 samples on one NextSeq500 high output flow cell with 151**×**2 pair-end run parameters according to manufactory manual and relying on default sequencing primers. Set both Index1 and Index2 as 8. Load at 1.8–2.2 pM concentration. (Figure 3)

***Optional:*** If the production sequencing doesn’t generate enough reads for some samples (i.e over 1M reads for samples enriched with the OM6 panel), another round of NextSeq could be conducted using the same library for these samples. Consider Hiseq or NovaSeq platforms for large scale projects.

## Expected outcomes

We expect to get and ∼150 bp precursors size and ∼110 bp probe size after digestion as shown in Figure1. The sequencing ready library size after size selection and purification should be ∼300 bp as detected by Tape Station and no/minimum primer dimmers 170–240, see Figure 2.

## Limitations

Poor quality of whole genome amplified genomic DNA may prevent hybridization, gap fill, and full library preparation. The protocol is plate-based, so the sample size is limited to a few thousand.

## Troubleshooting

### Problem 1

The sequencing library after size selection by Blue Pippin resulting DNA concentration is too low to load on Illumina sequencer. [Step 6d]

### Potential solution

Increase the pooling volume per sample from 2 ul to 5 ul for the Blue Pippin loading pool. Use the same elution volume 40 ul to increase the original DNA amount loaded in Blue Pippin.

### Problem 2

Primer dimers at 170–240 bp are still presenting in significant ratio to the desired library peak around 300 bp in diagnostic libraries detected by Tape Station after size selection by Blue Pippin.[Step 6e]

### Potential solution

Check the quality of single cell WGA DNA by size and concentration, make sure to use good quality WGA DNA for the majority of samples.

### Problem 3

Significant by product in large size more than 300 bp detected by Tape Station presented in probe production PCR.[Step 3 ]

### Potential solution

Check the template concentration used in production PCR, make sure to dilute it to 1 ng/ul; reduce the production PCR cycles to 10 or 11.

### Problem 4

Significant undigested probes ∼150 bp remains in the Tape Station quality control step.[Step 4]

### Potential solution

Check the concentration of the input precursor again to make sure <30 ng/ul concentration used in digestion reaction; With the same digestion setting, digest the probes again, and purify by Mini Elute, run quality control by Tape Station.

### Problem 5

Low sequencing quality presented by the illumina sequencer, including low passing filter clusters, low Q30. [Step 7]

### Potential solution

Consider the sequencing complexity in both the amplicon region and index region, especially when handling small panel (<100 targets) and small scale of samples (<20). Spike in 20% PhiX in such cases could help improve the overall sequencing quality.

## Resource availability

### Lead contact

Further information and requests for resources and reagents should be directed to and will be fulfilled by the lead contact: Ehud Shapiro: ehud.shapiro@weizmann.ac.il

### Materials availability

This study did not generate new unique reagents.

## Data Availability

The data supporting the current study are subject to the rules of regulations of the ethical committee of the Weizmann Institute of Sciences. Requests for data should be directed to the lead contact, Ehud Shapiro: ehud.shapiro@weizmann.ac.il For further details regarding the computational analysis, parallel execution, and the cell lineage system, please see: https://github.com/shapirolab/clineage
